# Diaqua­[5,5′-dicarb­oxy-2,2′-(propane-1,3-di­yl)bis­(1*H*-imidazole-4-carboxyl­ato)]manganese(II)

**DOI:** 10.1107/S1600536811012566

**Published:** 2011-04-07

**Authors:** Huai-Xia Yang, Xiaoli Zhou, Guanghua Jin, Xiang-Ru Meng

**Affiliations:** a Pharmacy College, Henan University of Traditional Chinese Medicine, Zhengzhou 450008, People’s Republic of China; b Experiment Administrative Center, Zhongzhou University, Zhengzhou 450044, People’s Republic of China; c Department of Chemistry, Zhengzhou University, Zhengzhou 450052, People’s Republic of China

## Abstract

The complex mol­ecule of the title compound, [Mn(C_13_H_10_N_4_O_8_)(H_2_O)_2_] or [Mn(H_4_pbidc)(H_2_O)_2_] (H_6_pbidc = 2,2′-(propane-1,3-di­yl)bis­(1*H*-imidazole-4,5-dicarb­oxy­lic acid), has 2 symmetry with the twofold rotation axis running through the Mn^2+^ cation and the central C atom of the propanediyl unit. The cation is six-coordinated by two N atoms and two O atoms from one H_4_pbidc^2−^ anion and two water O atoms in a considerably distorted octa­hedral coordination. In the crystal, adjacent mol­ecules are linked through O—H⋯O and N—H⋯O hydrogen bonds into a three-dimensional network.

## Related literature

For background to complexes based on 1*H*-imidazole-4,5-dicarb­oxy­lic acid, see: Ghosh *et al.* (2009 ▶); Liu *et al.* (2008 ▶); Sun & Yang (2007 ▶).
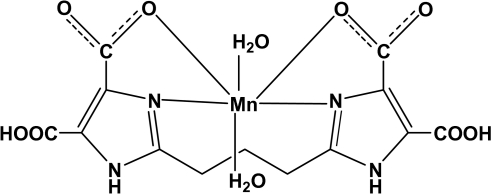


## Experimental

### 

#### Crystal data

[Mn(C_13_H_10_N_4_O_8_)(H_2_O)_2_]
*M* *_r_* = 441.22
Monoclinic, 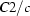

*a* = 15.620 (3) Å
*b* = 8.5310 (17) Å
*c* = 12.739 (3) Å
β = 97.07 (3)°
*V* = 1684.7 (6) Å^3^
*Z* = 4
Mo *K*α radiation
μ = 0.85 mm^−1^
*T* = 293 K
0.23 × 0.21 × 0.18 mm

#### Data collection

Rigaku Saturn diffractometer
Absorption correction: multi-scan (*CrystalClear*; Rigaku/MSC, 2006 ▶) *T*_min_ = 0.828, *T*_max_ = 0.862
3426 measured reflections
1464 independent reflections
1265 reflections with *I* > 2σ(*I*)
*R*_int_ = 0.026

#### Refinement

*R*[*F* ^2^ > 2σ(*F* ^2^)] = 0.037
*wR*(*F* ^2^) = 0.090
*S* = 1.09
1464 reflections
128 parameters
H-atom parameters constrained
Δρ_max_ = 0.47 e Å^−3^
Δρ_min_ = −0.45 e Å^−3^

### 

Data collection: *CrystalClear* (Rigaku/MSC, 2006 ▶); cell refinement: *CrystalClear*; data reduction: *CrystalClear*; program(s) used to solve structure: *SHELXS97* (Sheldrick, 2008 ▶); program(s) used to refine structure: *SHELXL97* (Sheldrick, 2008 ▶); molecular graphics: *XP* in *SHELXTL* (Sheldrick, 2008 ▶); software used to prepare material for publication: *SHELXL97*.

## Supplementary Material

Crystal structure: contains datablocks global, I. DOI: 10.1107/S1600536811012566/wm2475sup1.cif

Structure factors: contains datablocks I. DOI: 10.1107/S1600536811012566/wm2475Isup2.hkl

Additional supplementary materials:  crystallographic information; 3D view; checkCIF report

## Figures and Tables

**Table 1:** Selected bond lengths (Å)

Mn1—O5	2.107 (2)
Mn1—N1	2.237 (2)
Mn1—O1	2.3236 (19)

**Table 2:** Hydrogen-bond geometry (Å, °)

*D*—H⋯*A*	*D*—H	H⋯*A*	*D*⋯*A*	*D*—H⋯*A*
O5—H2*W*⋯O4^i^	0.85	1.94	2.780 (3)	168
O5—H1*W*⋯O3^ii^	0.85	1.93	2.780 (3)	174
N2—H2*A*⋯O4^iii^	0.86	1.97	2.785 (3)	159
O3—H3⋯O2	0.85	1.68	2.527 (3)	178

Symmetry codes: (i) 
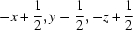
; (ii) 
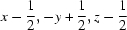
; (iii) 
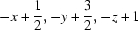
.

**Fig. 1.**
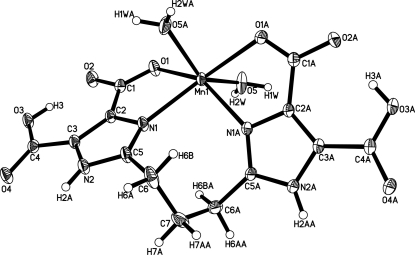
View of the title complex, showing the labelling of the atoms which are displayed with their displacement ellipsoids at the 30% probability level. [Symmetry code A: -x, y, -z + 1/2.]

## supplementary crystallographic information

### Comment

Up to date, a large number of metal-organic frameworks derived from 1*H*-imidazole-4,5-dicarboxylic acid (H_3_idc) have been synthesized since it is a good linker and can be successively deprotonated to generate H_2_idc^-^, Hidc^2-^ and idc^3-^ anions (Ghosh *et al.*, 2009; Liu *et al.*, 2008; Sun & Yang, 2007). Compared with H_3_idc, 2,2'-(1,3-propanediyl)bis-1*H*-imidazole-4,5-dicarboxylic acid (H_6_pbidc) can provide a greater tunability of structural frameworks because of the presence of the propanediyl spacer. However, complexes derived from this ligand have been scarcely reported. In this work, through the reaction of H_6_pbidc with MnSO_4_, we obtained the title complex [Mn(H_4_pbidc) (H_2_O)_2_], (I).

As shown in Figure 1, the Mn^2+^ cation in (I) is hexacoordinated and features a distorted octahedral coordination geometry. N1, O1, N1A, O1A atoms from the tetradentate H_4_pbidc^2-^ group coordinate to the cation in a chelating fashion and O5, O5A atoms from water molecules complete the coordination polyhedron. The entire complex molecule has symmetry 2. The bond angles around the Mn^2+^ cation significantly deviate from 90 or 180° (see supplementary material). Intramolecular O—H···O hydrogen bonds between the carboxyl/carboxylate groups stabilize the molecular configuration whereas O—H···O and N—H···O hydrogen bonds between the water molecules and carboxylate O atoms and between imidazole groups and carboxylate O atoms of adjacent molecules consolidate the crystal packing.

### Experimental

A mixture of MnSO_4_ (0.05 mmol), 2,2'-(1,3-propanediyl)bis-1*H*-imidazole-4,5-dicarboxylic (0.05 mmol), methanol (2 ml) and water (2 ml) was placed in a 25 ml Teflon-lined stainless steel vessel and heated at 433 K for 72 h, then cooled to room temperature. Light yellow crystals with good quality were obtained from the filtrate and dried in air.

### Refinement

H atoms were positioned geometrically and refined as riding atoms, with C—H = 0.97 Å, N—H = 0.86 Å and O—H = 0.85 Å, and with U_iso_(H) = 1.2 U_eq_(C,N,O).

### Crystal data

**Table:**

[Mn(C_13_H_10_N_4_O_8_)(H_2_O)_2_]	*F*(000) = 900
*M**_r_* = 441.22	*D*_x_ = 1.740 Mg m^−^^3^
Monoclinic, *C*2/*c*	Mo *K*α radiation, λ = 0.71073 Å
Hall symbol: -C 2yc	Cell parameters from 2738 reflections
*a* = 15.620 (3) Å	θ = 2.6–27.9°
*b* = 8.5310 (17) Å	µ = 0.85 mm^−^^1^
*c* = 12.739 (3) Å	*T* = 293 K
β = 97.07 (3)°	Prism, light yellow
*V* = 1684.7 (6) Å^3^	0.23 × 0.21 × 0.18 mm
*Z* = 4	

### Data collection

**Table:**

Rigaku Saturn diffractometer	1464 independent reflections
Radiation source: fine-focus sealed tube	1265 reflections with *I* > 2σ(*I*)
graphite	*R*_int_ = 0.026
Detector resolution: 28.5714 pixels mm^-1^	θ_max_ = 25.0°, θ_min_ = 2.6°
ω scans	*h* = −17→18
Absorption correction: multi-scan (*CrystalClear*; Rigaku/MSC, 2006)	*k* = −10→8
*T*_min_ = 0.828, *T*_max_ = 0.862	*l* = −15→12
3426 measured reflections	

### Refinement

**Table:**

Refinement on *F*^2^	Primary atom site location: structure-invariant direct methods
Least-squares matrix: full	Secondary atom site location: difference Fourier map
*R*[*F*^2^ > 2σ(*F*^2^)] = 0.037	Hydrogen site location: inferred from neighbouring sites
*wR*(*F*^2^) = 0.090	H-atom parameters constrained
*S* = 1.09	*w* = 1/[σ^2^(*F*_o_^2^) + (0.0405*P*)^2^ + 1.7694*P*] where *P* = (*F*_o_^2^ + 2*F*_c_^2^)/3
1464 reflections	(Δ/σ)_max_ < 0.001
128 parameters	Δρ_max_ = 0.47 e Å^−^^3^
0 restraints	Δρ_min_ = −0.45 e Å^−^^3^

### Special details

**Table:**

Geometry. All e.s.d.'s (except the e.s.d. in the dihedral angle between two l.s. planes) are estimated using the full covariance matrix. The cell e.s.d.'s are taken into account individually in the estimation of e.s.d.'s in distances, angles and torsion angles; correlations between e.s.d.'s in cell parameters are only used when they are defined by crystal symmetry. An approximate (isotropic) treatment of cell e.s.d.'s is used for estimating e.s.d.'s involving l.s. planes.
Refinement. Refinement of *F*^2^ against ALL reflections. The weighted *R*-factor *wR* and goodness of fit *S* are based on *F*^2^, conventional *R*-factors *R* are based on *F*, with *F* set to zero for negative *F*^2^. The threshold expression of *F*^2^ > σ(*F*^2^) is used only for calculating *R*-factors(gt) *etc*. and is not relevant to the choice of reflections for refinement. *R*-factors based on *F*^2^ are statistically about twice as large as those based on *F*, and *R*- factors based on ALL data will be even larger.

### Fractional atomic coordinates and isotropic or equivalent isotropic displacement parameters (Å^2^)

**Table:**

	*x*	*y*	*z*	*U*_iso_*/*U*_eq_	
Mn1	0.0000	0.22580 (7)	0.2500	0.0249 (2)	
N1	0.08997 (14)	0.4109 (3)	0.32272 (18)	0.0273 (5)	
N2	0.16454 (14)	0.5985 (3)	0.40995 (19)	0.0308 (6)	
H2A	0.1767	0.6876	0.4397	0.037*	
O1	0.13833 (12)	0.1209 (2)	0.26815 (16)	0.0336 (5)	
O2	0.27378 (12)	0.1518 (2)	0.34234 (17)	0.0382 (5)	
O3	0.35729 (12)	0.3642 (2)	0.44866 (16)	0.0336 (5)	
H3	0.3281	0.2937	0.4133	0.040*	
O4	0.34141 (12)	0.6152 (2)	0.48617 (17)	0.0370 (5)	
O5	0.00284 (13)	0.1299 (3)	0.09807 (16)	0.0466 (6)	
H1W	−0.0390	0.1314	0.0487	0.056*	
H2W	0.0460	0.1216	0.0637	0.056*	
C1	0.19523 (18)	0.2024 (3)	0.3170 (2)	0.0278 (6)	
C2	0.17401 (16)	0.3615 (3)	0.3491 (2)	0.0242 (6)	
C3	0.22101 (16)	0.4777 (3)	0.4038 (2)	0.0258 (6)	
C4	0.31248 (17)	0.4881 (3)	0.4495 (2)	0.0269 (6)	
C5	0.08645 (17)	0.5546 (3)	0.3616 (2)	0.0297 (7)	
C6	0.00932 (19)	0.6586 (4)	0.3507 (3)	0.0462 (9)	
H6A	0.0130	0.7285	0.4111	0.055*	
H6B	−0.0420	0.5947	0.3516	0.055*	
C7	0.0000	0.7559 (5)	0.2500	0.0562 (15)	
H7A	−0.0509	0.8214	0.2480	0.067*	

### Atomic displacement parameters (Å^2^)

**Table:**

	*U*^11^	*U*^22^	*U*^33^	*U*^12^	*U*^13^	*U*^23^
Mn1	0.0183 (3)	0.0268 (3)	0.0276 (3)	0.000	−0.0048 (2)	0.000
N1	0.0177 (11)	0.0278 (12)	0.0344 (13)	0.0007 (9)	−0.0048 (10)	−0.0048 (10)
N2	0.0222 (12)	0.0279 (12)	0.0396 (14)	−0.0003 (10)	−0.0068 (11)	−0.0085 (10)
O1	0.0237 (11)	0.0333 (11)	0.0416 (12)	0.0003 (9)	−0.0051 (9)	−0.0114 (9)
O2	0.0227 (11)	0.0373 (12)	0.0515 (13)	0.0084 (9)	−0.0078 (10)	−0.0074 (10)
O3	0.0208 (10)	0.0361 (12)	0.0408 (11)	−0.0011 (9)	−0.0094 (9)	−0.0059 (9)
O4	0.0236 (10)	0.0357 (12)	0.0490 (13)	−0.0055 (9)	−0.0062 (10)	−0.0099 (10)
O5	0.0241 (11)	0.0804 (17)	0.0328 (12)	0.0111 (11)	−0.0070 (10)	−0.0179 (11)
C1	0.0238 (15)	0.0319 (15)	0.0268 (14)	0.0015 (12)	−0.0008 (12)	−0.0008 (12)
C2	0.0174 (13)	0.0281 (14)	0.0262 (14)	0.0001 (11)	−0.0008 (12)	0.0002 (11)
C3	0.0191 (14)	0.0291 (14)	0.0278 (14)	−0.0010 (11)	−0.0028 (11)	−0.0012 (12)
C4	0.0206 (14)	0.0331 (16)	0.0261 (14)	−0.0018 (12)	−0.0002 (12)	0.0023 (12)
C5	0.0189 (14)	0.0298 (15)	0.0381 (16)	0.0004 (12)	−0.0054 (13)	−0.0094 (13)
C6	0.0235 (16)	0.0419 (18)	0.069 (2)	0.0065 (14)	−0.0091 (16)	−0.0271 (17)
C7	0.029 (2)	0.023 (2)	0.109 (5)	0.000	−0.020 (3)	0.000

### Geometric parameters (Å, °)

**Table:**

Mn1—O5	2.107 (2)	O3—H3	0.8500
Mn1—O5^i^	2.107 (2)	O4—C4	1.243 (3)
Mn1—N1	2.237 (2)	O5—H1W	0.8501
Mn1—N1^i^	2.237 (2)	O5—H2W	0.8500
Mn1—O1	2.3236 (19)	C1—C2	1.467 (4)
Mn1—O1^i^	2.3236 (19)	C2—C3	1.371 (4)
N1—C5	1.326 (3)	C3—C4	1.478 (4)
N1—C2	1.380 (3)	C5—C6	1.489 (4)
N2—C5	1.350 (3)	C6—C7	1.520 (4)
N2—C3	1.365 (3)	C6—H6A	0.9700
N2—H2A	0.8600	C6—H6B	0.9700
O1—C1	1.235 (3)	C7—C6^i^	1.520 (4)
O2—C1	1.303 (3)	C7—H7A	0.9700
O3—C4	1.269 (3)		
			
O5—Mn1—O5^i^	134.29 (13)	H1W—O5—H2W	101.9
O5—Mn1—N1	124.84 (9)	O1—C1—O2	122.4 (3)
O5^i^—Mn1—N1	88.68 (8)	O1—C1—C2	119.2 (2)
O5—Mn1—N1^i^	88.68 (8)	O2—C1—C2	118.4 (2)
O5^i^—Mn1—N1^i^	124.84 (9)	C3—C2—N1	109.7 (2)
N1—Mn1—N1^i^	90.22 (11)	C3—C2—C1	133.2 (2)
O5—Mn1—O1	79.48 (8)	N1—C2—C1	117.1 (2)
O5^i^—Mn1—O1	83.31 (8)	N2—C3—C2	105.4 (2)
N1—Mn1—O1	72.64 (7)	N2—C3—C4	122.2 (2)
N1^i^—Mn1—O1	147.34 (8)	C2—C3—C4	132.4 (2)
O5—Mn1—O1^i^	83.31 (8)	O4—C4—O3	123.7 (2)
O5^i^—Mn1—O1^i^	79.48 (8)	O4—C4—C3	119.3 (2)
N1—Mn1—O1^i^	147.34 (8)	O3—C4—C3	117.0 (2)
N1^i^—Mn1—O1^i^	72.64 (7)	N1—C5—N2	110.5 (2)
O1—Mn1—O1^i^	134.71 (10)	N1—C5—C6	125.9 (2)
C5—N1—C2	105.8 (2)	N2—C5—C6	123.5 (2)
C5—N1—Mn1	138.97 (18)	C5—C6—C7	113.4 (3)
C2—N1—Mn1	114.56 (17)	C5—C6—H6A	108.9
C5—N2—C3	108.6 (2)	C7—C6—H6A	108.9
C5—N2—H2A	125.7	C5—C6—H6B	108.9
C3—N2—H2A	125.7	C7—C6—H6B	108.9
C1—O1—Mn1	115.86 (17)	H6A—C6—H6B	107.7
C4—O3—H3	109.4	C6^i^—C7—C6	113.7 (3)
Mn1—O5—H1W	124.9	C6^i^—C7—H7A	107.4
Mn1—O5—H2W	127.8	C6—C7—H7A	109.3
			
O5—Mn1—N1—C5	121.0 (3)	O2—C1—C2—C3	−0.1 (5)
O5^i^—Mn1—N1—C5	−92.4 (3)	O1—C1—C2—N1	0.3 (4)
N1^i^—Mn1—N1—C5	32.5 (3)	O2—C1—C2—N1	−178.9 (2)
O1—Mn1—N1—C5	−175.8 (3)	C5—N2—C3—C2	0.6 (3)
O1^i^—Mn1—N1—C5	−24.3 (4)	C5—N2—C3—C4	−179.9 (2)
O5—Mn1—N1—C2	−69.7 (2)	N1—C2—C3—N2	−0.2 (3)
O5^i^—Mn1—N1—C2	76.92 (19)	C1—C2—C3—N2	−179.0 (3)
N1^i^—Mn1—N1—C2	−158.2 (2)	N1—C2—C3—C4	−179.5 (3)
O1—Mn1—N1—C2	−6.49 (17)	C1—C2—C3—C4	1.6 (5)
O1^i^—Mn1—N1—C2	144.92 (17)	N2—C3—C4—O4	−6.9 (4)
O5—Mn1—O1—C1	138.8 (2)	C2—C3—C4—O4	172.4 (3)
O5^i^—Mn1—O1—C1	−83.7 (2)	N2—C3—C4—O3	173.2 (3)
N1—Mn1—O1—C1	7.01 (19)	C2—C3—C4—O3	−7.5 (4)
N1^i^—Mn1—O1—C1	68.3 (3)	C2—N1—C5—N2	0.8 (3)
O1^i^—Mn1—O1—C1	−151.7 (2)	Mn1—N1—C5—N2	170.6 (2)
Mn1—O1—C1—O2	172.9 (2)	C2—N1—C5—C6	178.2 (3)
Mn1—O1—C1—C2	−6.3 (3)	Mn1—N1—C5—C6	−11.9 (5)
C5—N1—C2—C3	−0.4 (3)	C3—N2—C5—N1	−0.9 (3)
Mn1—N1—C2—C3	−173.07 (18)	C3—N2—C5—C6	−178.5 (3)
C5—N1—C2—C1	178.7 (2)	N1—C5—C6—C7	−87.0 (4)
Mn1—N1—C2—C1	6.0 (3)	N2—C5—C6—C7	90.1 (4)
O1—C1—C2—C3	179.1 (3)	C5—C6—C7—C6^i^	60.5 (2)

Symmetry codes: (i) −*x*, *y*, −*z*+1/2.

### Hydrogen-bond geometry (Å, °)

**Table:**

*D*—H···*A*	*D*—H	H···*A*	*D*···*A*	*D*—H···*A*
O5—H2W···O4^ii^	0.85	1.94	2.780 (3)	168
O5—H1W···O3^iii^	0.85	1.93	2.780 (3)	174
N2—H2A···O4^iv^	0.86	1.97	2.785 (3)	159
O3—H3···O2	0.85	1.68	2.527 (3)	178

Symmetry codes: (ii) −*x*+1/2, *y*−1/2, −*z*+1/2; (iii) *x*−1/2, −*y*+1/2, *z*−1/2; (iv) −*x*+1/2, −*y*+3/2, −*z*+1.
